# Outcomes of the combined lifestyle intervention CooL during COVID-19: a descriptive case series study

**DOI:** 10.1186/s12889-023-17501-x

**Published:** 2024-01-02

**Authors:** Ester Janssen, Nicole Philippens, Stef Kremers, Rik Crutzen

**Affiliations:** 1 Department of Health Promotion, NUTRIM, School of Nutrition and Translational Research in Metabolism, Maastricht University, Maastricht, The Netherlands; 2 Department of Health Promotion, CAPHRI, Care & Public Health Research Institute, Maastricht University, Maastricht, The Netherlands

**Keywords:** Lifestyle, CooL, Overweight, Obesity, COVID-19, Intervention, Effect, (Perceived) health

## Abstract

**Background:**

The main objective of this nationwide study was to investigate changes in outcomes between baseline and eight months of participation regarding anthropometrics, control and support, physical activity, diet attentiveness, perceived fitness, sleep, and stress of participants in Coaching on Lifestyle (CooL), a Combined Lifestyle Intervention (CLI). Since the study took place when the COVID-19 pandemic emerged, we defined a subobjective, i.e., to address changes in intervention outcomes over time while participants were exposed to pandemic-related restrictions and uncertainties.

**Methods:**

Data were collected from November 2018 until October 2021 at different locations across the Netherlands from 1824 participating adults, meeting the CLI inclusion criteria. We collected a broad set of data on anthropometrics (weight, body mass index (BMI), waist circumference), control and support (self-mastery, social support), physical activity (sedentary time on least/most active days, physical active minutes), diet attentiveness (attentiveness to meal composition, awareness to amounts of food and attentiveness to consuming), alcohol consumption, smoking, perceived fitness (perceived health, fitness when waking, fitness during daytime, impact daily stress), sleep and stress.

**Results:**

All outcomes showed improvements after eight months compared to baseline except for social support and smoking. Large effect sizes were found on weight (0.57), waist circumference (0.50) and perceived health (0.50). Behaviour patterns showed small to large effect sizes, with the largest effect sizes on diet attentiveness (i.e., attentiveness to meal composition (0.43), awareness to amounts of food (0.58) and attentiveness to consuming (0.39)). The outcomes of participants pre COVID-19 versus during COVID-19 showed differences on self-mastery (p = 0.01), sedentary time (all underlying constructs p < 0.02), perceived fitness (all underlying constructs p < 0.02) and stress (p < 0.01).

**Conclusion:**

The results show that small changes in multiple behaviours go along with a large positive change in perceived health and health-related outcomes in line with the lifestyle coaching principles. In addition, participating in CooL may have protected against engaging in unhealthier behaviour during the pandemic.

**Trial registration:**

As the CLI is considered usual health care that does not fall within the scope of the Dutch Medical Research Involving Human Subjects Act, this study was exempt from trial registration.

## Background

In 2021, 50% of Dutch people aged 18 and older, were overweight and approximately 14% were obese [1]. Obesity is considered a disease according to the World Health Organisation [2] and the Dutch Health council [3]. Furthermore, obesity is associated with an increased risk for many other diseases, such as diabetes mellitus type 2, cardiovascular disease, various cancers [4–6], mental health problems (e.g., depression) [7] and a diminished quality of life [8]. Consensus has been reached internationally [9] on the importance of an integrated approach to target overweight and obesity, including limited energy intake, healthy food choices and regular physical activity. The Dutch national guidelines added stress management and sleep as essential elements to tackle overweight and obesity [10].

As of January 2019, Combined Lifestyle Interventions (CLIs) are part of basic health insurance in the Netherlands. A CLI is an intervention for people with overweight or obesity, stimulating weight reduction by promoting sustained healthier behaviour. In the intervention, participants are coached towards a healthier lifestyle. The CLIs exist of a combination of group and individual sessions and cover at least the topics of healthy diet, physical activity and behavioural change. Based on the Dutch national guidelines on the treatment of obesity and the relationship between stress and obesity and sleep and obesity, both lifestyle themes are also considered an essential part of the CLI [11, 12].

The inclusion criteria for CLIs in the Netherlands are: [1] being 18 or older; (2a) having a Body Mass Index (BMI) between 25 and 30 kg/m2 in combination with a waist circumference over 88 cm for women or over 102 cm for men, or with comorbidity (increased risk of) diabetes or cardiovascular disease, osteoarthritis or sleep apnea), or (2b) having a BMI > 30 kg/m2 regardless of waist size or comorbidity; and [3] being sufficiently motivated to complete the two-year intervention as judged by the referrer (e.g., the general practitioner or practice nurse) and the CLI-coach.

The Coaching on Lifestyle intervention (CooL) is one of six CLIs that are approved by the Dutch Institute for Public Health and Environment (in Dutch: RIVM) for being effective in facilitating weight reduction. The intervention has two phases: an intensive behavioural change phase of eight months, followed by a less-intensive 16-month behavioural maintenance phase summing up to a total duration of two years. Baseline measurements are done during intake, followed by measurements after the behavioural change phase (8 months) and after the behavioural maintenance phase (24 months). So far, research on the CLI has been done on Slimmer [13] and Beweegkuur [14] and on CooL in a regional setting: the CooL-pilot and the healthyLIFE study [15, 16]. All CLI’s are showing comparable weight loss as well as additional benefits in positive health [16], metabolic risk factors [13, 14] and/or health related behaviour [13–16]. The main objective of the present nationwide study is to look at the changes in outcomes of participants in the behavioural change phase of the CooL-intervention on the topics of anthropometrics (weight, BMI, waist circumference), control and support (self-mastery, social support), physical activity (sedentary time on least/most active days, physical active minutes), diet attentiveness (attentiveness to meal composition, awareness to amounts of food and attentiveness to consuming), alcohol consumption, smoking, perceived fitness (perceived health, fitness when waking, fitness during daytime, impact daily stress), sleep and stress (see Table 1).

The COVID-19 pandemic entered the Netherlands in February 2020, resulting in stringent COVID-19 measures that came into effect from March 2020 onwards. Obesity is considered a risk factor for a COVID-19 infection but also a risk factor for a more severe disease course resulting in higher mortality rates [17, 18]. Both Dutch and international studies found that 70–90% of all COVID-19 patients admitted to Intensive Care Units with respiratory failure, were overweight [19, 20]. The immune system of patients with obesity is less capable of fighting viruses and bacteria. Lifestyle improvements lead to improvements in the immune system [21], which might be an additional reason for deployment of the CLI for overweight people.

We expected the pandemic, and the measures aimed to curb it, to have an impact on the CLI-participants [22]. As the severity of the disease course increased for overweight patients, it led to more stress in this high-risk population [23]. The COVID-19 pandemic resulted for some people in a higher sense of urgency to start with a weight reduction program. Others, on the other hand, were hesitant to attend group meetings due to their high-risk profile related to a potential COVID-19 infection. The consequences of the COVID-19 restrictions such as a temporary curfew, closing (sports) facilities, working at home and wearing face masks in public areas led to feelings of loneliness but also impacted lifestyle routines [23]. In addition, CLIs were initially temporarily suspended, pending guidelines on restricted human contact. Some CLI-groups were permanently closed, others restarted in digital modus, providing additional challenges for both coaches and participants. COVID-19 shifted priority for caretakers and participants as there were shortages of staff due to sickness or deployment in more critical roles, impacting availability and attendance of (digital) CLI-sessions [24].

Therefore, the subobjective of this study was to investigate the effect of COVID-19 implications and restrictions on the intervention outcomes.

## Methods

### CooL-intervention

The CooL-intervention aims for higher perceived quality of life, healthier eating habits (including a focus on healthy food choices, food quantities and eating with attention), more physical activity, less sedentary behaviour, attention for high quality sleep and relaxation, and positive changes in physical outcomes such as weight, BMI and waist circumference. CooL includes an intake (1 h), a behavioural change phase of eight months (phase 1) with a follow-up phase of sixteen months (phase 2). The intervention consists of a combination of individual sessions (six hours in total, divided in 6 to 12 sessions depending on the preferences of the participant and coach) and 16 group sessions (1, 5 h each) all led by one and the same coach. Phase 1 and phase 2 both include eight group sessions with a higher density of sessions in phase 1 compared to phase 2 [15].

The CooL-intervention is an open CLI, which means that CooL has no strict protocol. Instead, it allows CooL-coaches to adapt the intervention to their target audience and context, within certain boundaries and restrictions. Participants pursue a predefined set of final objectives on knowledge and skills, supported by the coach who secures the main effective elements (e.g., goal setting, mobilizing social support, modelling, self-management and self-monitoring) of the CooL-intervention in implementation [15]. The CooL-coaches are trained and licensed professionals who coach participants to take responsibility for their personal lifestyle changes by addressing motivation, personal objectives and behavioural change. Participants are stimulated and supported towards more self-steering and self-management by identifying, mapping and putting personal health related behaviour into action. The main objective is to coach and activate participants to a sustained healthier lifestyle in line with their individual needs and goals.

### CooL-intervention during COVID-19

The COVID-19 implications and restrictions resulted in adaptations in the way CooL was offered to participants. Some participants finalized the first eight months of CooL completely, before COVID-19 broke out in the Netherlands, others participated in CooL during the COVID-19 pandemic and measures. The first infection was detected in the Netherlands on February 27th, 2020, the first regional restrictions were imposed on March 6th and the ‘intelligent lockdown’ (a semi-lockdown with free human movement but restricted human contact) was introduced as of March 23th [25]. We used a cut-off date of April 1st, 2020, as participants finishing phase 1 of CooL before this date will have suffered limited to no impact on their lifestyle which cannot be guaranteed for participants finishing phase 1 of CooL after April 1st, 2020. By means of the cut-off date we distinguished between participants that were potentially impacted by COVID-19 while participating in CooL and participants that were not impacted by COVID-19.

The way in which CooL was offered, changed during the COVID-19 pandemic. These changes were inventoried by an additional survey among CooL-coaches and by adding questions related to COVID-19 to the existing CooL-questionnaire.

The open character of CooL provided ample opportunity for CooL-coaches to make adaptations to the content of the intervention, e.g., providing room for pressing topics like COVID-19 or COVID-19-related stress. In addition, the temporary expansion in the CLI-regulations in terms of health insurance coverage made it possible to offer CooL digitally instead of via face-to-face contact only [26].

Observations from daily practice showed that COVID-19 resulted in higher dropout rates, resulting in financial consequences for the coaches and motivational challenges for the remaining group members and the coach. Some CooL-coaches completely quit executing CooL due to uncertainty, loss of motivation and/or resistance to online coaching thereby leaving their participants no other option than to quit CooL. Others decided to start up CooL, as COVID-19 caused an income drop for self-employed coaches and the CLI offered a basic and stable income. This observed impact of COVID-19 on coaches and participants of CooL, gave rise to the initiation of this subobjective.

### Study design and population

As CooL is part of regular health care, a control group receiving no treatment would be unethical, making a descriptive case series study the most appropriate study design in the Dutch context. The participants, all Dutch-speaking adults living in the Netherlands, were included from November 2018 until October 2021 at different locations throughout the Netherlands. Almost all participants met the inclusion criteria for participating in a CLI. In some cases (n = 5, 0.3%), BMI at baseline was below the inclusion threshold, potentially due to lifestyle changes in the time between participant’s application and the start of CooL. Since the waist circumference of these participants was above the threshold for inclusion, these cases were included.

### Data collection

We used a questionnaire and anthropometric measurements to collect a broad set of data. The questionnaire was partly based on existing validated questionnaires [27–29], and partly based on input from a focus group session with the Dutch Association of Lifestyle coaches (BLCN) to define questions that match the scope and working method of the lifestyle coach with a strong focus on manageability of the questionnaire, as CooL is part of basic healthcare. The outcome measures we collected can be divided into the categories anthropometrics (i.e., weight/BMI and waist circumference), control and support (i.e., self-mastery and social support), physical activity (i.e., sedentary time on least/most active days and active minutes), diet attentiveness (attentiveness to meal composition, awareness to amounts of food and attentiveness to consuming), alcohol use and smoking, perceived fitness (i.e., perceived health, perceived fitness when waking, perceived fitness during daytime and impact of stress on daily functioning), sleep and stress.

**Table 1:** Different constructs per outcome category

Category	Constructs	Contributing to
anthropometrics	weight, body mass index (BMI), waist circumference	weight management
control and support	self-mastery, social support	self-management thereby improving quality of life
physical activity	sedentary time least/most active days, physical active minutes	awareness and perception of behaviour (physical activity)
diet attentiveness, alcohol use and smoking	attentiveness to meal composition, awareness to amounts of food and attentiveness to consuming, alcohol consumption, smoking	awareness and perception of behaviour (diet, alcohol use, smoking)
perceived fitness	perceived health, fitness when waking, fitness during daytime, impact daily stress	awareness and perception of personal fitness, thereby improving quality of life
sleep	sleep	awareness and perception of behaviour (sleep)
stress	stress	awareness and perception of behaviour (stress)

During the course of the study, the questionnaire was adjusted with textual simplifications in both questions and answers preserving the original essence as much as possible and extended with additional questions covering changes in context (e.g., COVID-19). We collected information on the initiation of CooL during COVID-19, i.e., a digital start or a physical (face-to-face) start, and on the continuation mode of the sessions.

Data were collected at three time points during the CooL-intervention: at the beginning of the intervention, during the intake (T0); after 8 months, at completion of phase 1 of the intervention (T1); and after 24 months, at completion of the intervention (T2). Data from T2 were not yet available at the time of the analysis and are not presented in this article.

### Demographics

At baseline, participants were asked to report their personal characteristics such as gender, date of birth, country of birth and highest completed education, marital status, living situation and occupational status. Educational level was categorized as low (i.e., no education, primary education or junior secondary education), intermediate (e.g., senior secondary education) and high (e.g., higher professional and vocational education or university) according to the definitions of the Dutch Central Bureau of Statistics [30]. The living situation was divided into living together with someone (married or cohabiting) with or without kids and living alone (divorced, unmarried, or widowed) with or without kids. The occupational status was categorized as: working (paid work, voluntary work or self-employed) and not working (homemaker, unemployed/job seeker, retired/in early retirement, disabled or student). Country of birth was categorized into Dutch or non-Dutch.

### Anthropometrics

Normally anthropometric data (weight, length and waist circumference) are being measured by the CooL-coaches with professional equipment according to the guidelines provided by the Dutch Association of General Practitioners (Dutch: Nederlands Huisartsen Genootschap, NHG) [31]. Body weight (kg) was measured in kilogram, rounded off the nearest decimal. Height (m) was measured to the nearest centimetre without shoes. Waist circumference measurements were obtained to the nearest centimetre with a tape measure.

### Control and support

Changes in self-management, of which self-mastery is an important aspect, are related to changes in quality of life and self-efficacy [32]. Self-mastery is defined by Pearlin as the extent to which one regards one’s life-chances as being under one’s own control in contrast to being fatalistically ruled [33]. The self-mastery questions in the questionnaire were based on the short version of the Pearlin Mastery Scale using four questions (for example “I have little control over the things that happen to me”) and a 5-point Likert scale ranging from strongly agree (1) to strongly disagree (5) [ 27]. To identify social support, we questioned the perceived support of close ones using a 5-point Likert scale ranging from no support at all (1) to a lot of support (5).

### Physical activity

The outcome measurements on physical activity, diet and perceived fitness were defined in cooperation with the BLCN with the objective to capture the essence and map the desired outcomes of lifestyle coaching in a minimum set of questions. Physical activity was assessed with questions on sedentary behaviour, both on most and least active days (“What is the average number of hours you spent sitting on the day of the week you sit the most?”) and the number of physical activity minutes per day (“What is the average minutes per day that you are physically active (in minimum bouts of 10 minutes)?”).

### Diet attentiveness, alcohol use and smoking

We defined questions on diet attentiveness, in line with the input of the BLCN, based on the knowledge that deliberate behaviour changes start with awareness. We used questions on the awareness of participants towards meal composition (How much attention do you usually pay to what you eat?) and meal quantities (How aware are you usually of the amount you eat?) and awareness during the actual consumption of food (With how much attention do you usually eat?) using a 5-point Likert scale from very little attention (1) to a lot of attention (5). In addition, we asked the number of units of alcohol consumed and units smoked per day.

### Perceived fitness

Perceived fitness existed of questions, in line with the input of the BLCN, on perceived fitness when waking up and during the day, the impact of stress on daily functioning and on perceived health (i.e., feeling good about oneself, the extent of self-care invested and the perception of one’s general health). Questions were answered using a 5-point Likert scale, ranging from not good at all (1) to very good (5).

### Sleep

We defined a specific set of questions around the sub-constructs: subjective sleep quality, sleep latency, sleep duration, habitual sleep efficiency, sleep disturbances, use of sleep medication and daytime dysfunction, analogous to the validated and widely used PSQI-questionnaire [28]. Each subconstruct was covered by one or two question(s) using a numerical value or a 4-point Likert scale, ranging from ‘never’ (1) to ‘three times per week or more frequently’ (4).

### Stress

For stress, the validated Perceived Stress Scale questionnaire was used, which exists of ten questions using a 5-point Likert scale from never (1) to always (5) [ 29].

### COVID-19

We used a brief survey for the lifestyle coach in retrospect to collect data on the way CooL was offered during COVID-19. The questions were related to the start date of the intervention derived from the date of the intake, the way the intervention was offered and the mode in which the intervention was started (e.g., starting in face-to-face mode versus digital mode). We used four categories to distinguish the way the intervention was offered: face-to-face sessions only, digital sessions only, a combination with more face-to face than digital sessions and a combination with more digital than face-to-face sessions.

### Analyses

As a first step, we recoded some of the variables to facilitate interpretation in the sense that a higher/positive score refers to a desirable trend and a lower/negative score to an undesirable trend in the variable. For constructs based on validated questionnaires (i.e., self-mastery, sleep and stress) we adopted the accompanying approach. Secondly, we performed an exploratory factor analysis using R software and calculated McDonald’s omega to assess the internal structure of items regarding the constructs perceived health (T0: 0.66, T1: 0.75), self-mastery (both T0 and T1: 0.71), sleep (T0: 0.76, T1: 0.74), stress (T0: 0.87, T1: 0.88) and motivation (T0: 0.25). These analyses justified summarizing the lifestyle constructs by item score means for all, except the construct motivation.

For all items and constructs, we ran descriptive analyses (e.g., means, standard deviations). Changes in outcome measures over time were analysed using paired t-tests (T1 versus T0). Effect sizes were calculated using Cohen’s d and the outcomes were interpreted in accordance with Lipsey’s guidelines for each pair of outcomes, i.e., an effect size smaller than or equal to 0.32 is considered small, an effect size between 0.33 and 0.55 is considered medium and an effect size of 0.56 or above is considered large [34]. To improve comprehensibility effect sizes are represented such that positive values represent change in the desired direction whereas negative values represent change in the undesired direction.

To be considered successful, the target for the CLI (including CooL) is a 5% weight loss after the two years intervention, as set by the Dutch Healthcare Institute (Dutch: Zorginstituut) based on the guidelines set up by the Dutch Institute for Quality in Health Care (Dutch: CBO) as well as their English counterpart (NICE) [35]. The data in this study covers the first phase of CooL only (8 months), still leaving 16 months to further extend weight loss. We categorized the outcomes on weight: 5% weight loss or more, between 0 and 5% weight loss, weight stabilization or weight gain to map the percentage of participants with weight loss.

Next, we split the dataset in two subgroups: we used the cut-off date of April 1st, 2020, to distinguish between the subgroups pre-COVID and during-COVID. The cut-off date was based on the date the intervention started and derived from that, the date the participants finished phase 1 of CooL. This distinction enabled comparison of differences from T0 to T1 between participants that were potentially impacted by COVID-19 and those that were not impacted by COVID-19. For all these differences we performed independent T-tests comparing subgroups. All T-tests were performed using SPSS- software (version 27). We used a threshold value of p = 0.05 for all t-tests. Missing data were excluded from the statistical analyses because these cases could not be included in the calculation of the differences between T0 and T1.

To explore the assumption that small behavioural changes sum up to medium and large effects in anthropometrics, a post-hoc sensitivity analysis was performed to compare the trend in changes (desired, neutral or undesired) in behaviour components to the trend in changes (desired, neutral or undesired) in the outcome components weight/BMI and waist circumference.

### Ethics

This study was submitted to and approved by the Research Ethics Committee of the Faculty of Health, Medicine and Life Sciences of Maastricht University (FHML-REC/2019/073). All participants gave their informed consent for their anonymised personal data to be used for research purposes.

## Results

### Participants demographics

**Fig. 1:**
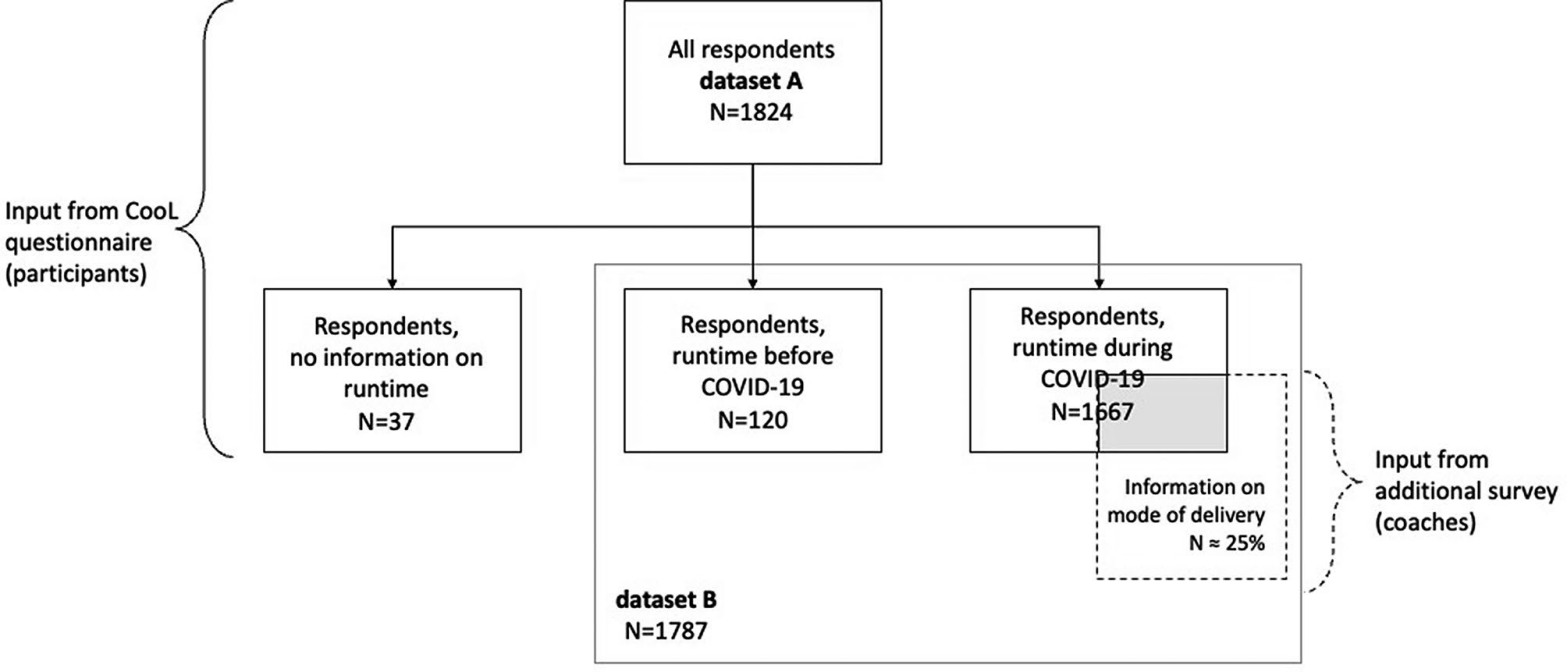
Flowchart of study participants

A total of 1824 adults participated between November 2018 and October 2021 (dataset A, see Fig. 1).

**Table 2:** Demographics of the participants (N = 1824)

Category	Demographic	Percentage of participants in dataset*
Gender	Male	28%
	Female	72%
Age	Until 35 years	11%
	35–44 years	16%
	45–54	29%
	55–64 years	28%
	65+	16%
Living situation	Single	18%
	Single parent	7%
	Living together with kids	43%
	Living together without kids	28%
	Other	4%
Country of birth	Dutch	95%
	Non-Dutch	5%
Working situation	Employed	75%
	Unemployed	25%
Education	Lower level	28%
	Intermediate level	39%
	Higher level	33%

*Amount of missing data differs per item per measurement moment (range 3.3–7.2%)

Of all participants a total of 28% were male and 72% female. This ratio is in line with the data from the national CLI-monitor [36]. Most participants (95%) were born in the Netherlands. Two third of the participants had a lower or intermediate level of education; 25% did not have a steady job (anymore) and over 70% of the participants were living together with a partner (Table 2).

### Subgroups cool during COVID-19

We defined subgroups of participants that were potentially impacted by COVID-19 and participants that were not (see Table 3). A total of 120 participants (7%) finished phase 1 before April 1st, 2020. Most participants (n = 1667, 91%) finished the first phase of CooL after April 1st, 2020, which implies that those participants were potentially affected by the COVID-19 implications and measures when participating in CooL. Both subgroups of respondents are included in dataset B (see Fig. 1).

From roughly a quarter of the participants (24%) with a runtime during COVID-19 we received information from the coaches on the way CooL was offered during COVID-19 by means of an additional survey (see Fig. 1). 80% of these participants started in face-to-face (physical) mode whereas 20% started digitally. Almost all participants received the individual and group sessions in CooL through a combination of physical and digital mode. Most participants received more physical than digital sessions (83% of the physical starters, 52% of the digital starters), followed by more digital than physical sessions (14% of the physical starters, 47% of the digital starters), 2% of all participants received physical sessions only and 0.5% digital sessions only.

**Table 3:** Number and percentage of participants per subgroup

Subgroup	N	Percentage of total
Runtime pre-COVID (T1 before April 1st, 2020)	120	7%
Runtime during COVID (T1 after April 1st, 2020)	1667	91%
No information on runtime	37	2%
TOTAL	1824	100%

### Results on all items and constructs

In the result section all outcomes and effect sizes on the complete dataset (A) are mentioned. When comparing the outcomes of participants pre and during COVID-19 (dataset B) significant findings are mentioned in the text with the corresponding p-value. Table 4 displays all outcome measurements both on dataset A and dataset B including mean values and standard deviations as well as confidence intervals on changes in outcomes.

**Table 4:** Results on all outcome measurements from dataset A and B

		Full sample (N = 1824) – dataset A				Pre-COVID-19 (n = 120) versus during COVID-19 (n = 1667) - dataset B		
Category	Construct/factor	T0 M (sd)	T1 M (sd)	∆ T0-T1 [95% CI]	Effect size T0-T1**	Pre COVID- ∆ TOT1 (sd)	During COVID- ∆ TOT1 (sd)	T-test Pre versus During COVID on ∆ T0T1 (p-value)
Anthropometrics	Weight	106.38 (18.50)	103.01 (18.37)	-3.44 [-3.73; -3.15]*	0.57	-3.68 (6.71)	-3.45 (5.85)	0.98
	BMI	35.97 (5.49)	34.80 (5.58)	-1.16 [-1.26; -1.06]*	0.58	-1.29 (2.40)	-1.17 (1.96)	0.92
	Waist circumference	116.30 (13.24)	112.35 (14.27)	-4.21 [-4.65; -3.77]*	0.50	-3.83 (5.93)	-4.23 (8.50)	0.27
Control & support	Self-mastery	2.54 (0.75)	2.48 (0.69)	-0.07 [-0.11; -0.03]*	0.10	-0.29 (0.74)	-0.06 (0.70)	0.01*
	Social support	3.77 (0.94)	3.73 (0.90)	0.03 [-0.02; 0.08]	0.03	0.15 (1.01)	-0.04 (0.93)	0.06
Physical activity	Sedentary time (least active)	9.56 (3.50)	8.77 (3.36)	-0.81 [-0.98; -0.65]*	0.25	0.05 (4.45)	-0.83 (3.16)	0.02*
	Sedentary time (most active)	6.31 (3.40)	5.79 (3.14)	-0.57 [-0.74; -0.41]*	0.18	0.41 (4.41)	-0.64 (3.10)	0.01*
	Physical active minutes	95.24 (112.04)	108.37 (111.13)	14.56 [9.33; 19.79]*	0.15	8.97 (96.54)	14.91 (99.40)	0.63
Diet attentiveness, alcohol and smoking	Attentiveness to meal composition	3.11 (0.98)	3.53 (0.83)	0.43 [0.38; 0.48]*	0.43	0.56 (0.92)	0.43 (1.00)	0.26
	Awareness of amounts of food	2.81 (0.97)	3.43 (0.83)	0.62 [0.57; 0.68]*	0.58	0.77 (0.97)	0.62 (1.10)	0.18
	Attentiveness to consuming	2.84 (1.00)	3.25 (0.91)	0.41 [0.36; 0.46]*	0.39	0.29 (1.07)	0.41 (1.03)	0.28
	Alcohol consumption	1.41 (2.02)	1.04 (1.59)	-0.32 [-0.41; -0.25]*	0.19	-0.10 (1.45)	-0.34 (1.68)	0.12
	Smoking	0.75 (3.61)	0.68 (3.24)	-0.05 [-0.15; 0.06]	0.02	-0.21 (2.24)	-0.05 (2.18)	0.45
Perceived fitness	Perceived health	9.07 (2.22)	10.21 (2.07)	1.15 [1.04; 1.26]*	0.50	1.81 (2.63)	1.12 (2.26)	0.01*
	Fitness (when waking)	2.38 (0.91)	2.63 (0.80)	0.26 [0.22; 0.31]*	0.28	0.54 (0.97)	0.25 (0.95)	0.01*
	Fitness (during daytime)	2.47 (0.85)	2.65 (0.79)	0.18 [0.14; 0.23]*	0.19	0.45 (1.03)	0.17 (0.97)	0.01*
	Impact stress (daily)	2.21 (0.94)	2.24 (0.89)	0.05 [0.00; 0.09]*	0.05	0.28 (0.90)	0.03 (0.95)	0.02*
Sleep	Sleep (summary)	7.13 (4.12)	6.09 (3.81)	-1.10 [-1.30; -0.91]*	0.30	-1.44 (3.22)	-1.10 (3.70)	0.42
Stress	Stress (summary)	14.24 (6.67)	13.07 (6.30)	-1.37 [-1.69; -1.05]*	0.23	-3.48 (6.48)	-1.36 (5.80)	0.01*

*p < 0.05

**Positive effect size representing an effect in desired direction, negative effect size representing an effect in undesired direction

### Anthropometrics

Weight, BMI and waist circumference all showed a decrease after eight months (T1) compared to baseline (T0). The BMI of the participants was on average 35.97 at T0 and decreased with 1.16 BMI-points at T1. The average weight loss was 3.44 kg at T1, corresponding to a 3.2% average weight loss per participant after eight months. In total 72% of the participants lost weight. 29% lost more than 5% at T1. The average waist circumference of the participants decreased from 116.3 cm at T0 to 112.4 cm at T1. The change in waist circumference demonstrated a medium effect size (0.50) at T1, whereas weight and BMI and showed a large effect size at T1 (0.57 and 0.58 respectively).

Participation in CooL during versus pre COVID-19 did not show a significant difference on weight, BMI or waist circumference of the participants.

### Control and support

Self-mastery showed a decrease at T1 compared to baseline with a small effect size (0.10) in the desired direction. Social support showed no change over time.

Differences in outcomes between participants pre versus during COVID-19 were present for self-mastery with a bigger change for participants pre COVID-19 (p = 0.01).

### Physical activity

Sedentary time decreased at T1 both for least and most active days of the week: participants spent on average 49 min less sitting on least active and 34 min less sitting on most active days compared to baseline. The average daily active minutes (in minimum bouts of 10 min) increased from 95 min at T0 to on average 108 min at T1. The effect size on both sedentary (0.25 for least active days and 0.18 for most active days) and active time (0.15) was small.

Comparing the outcomes pre and during COVID-19: participants during COVID-19 showed a decrease in sedentary time compared to baseline whereas participants pre COVID-19 showed a small increase for both least active and most active days (p < 0.02). No difference between both subgroups could be detected on physical active minutes.

### Diet attentiveness, alcohol and smoking

Over time the participants showed an increase in attentiveness for meal composition, awareness for the amounts of food selected and attentiveness when consuming food. In addition, the participants showed a decrease in alcohol consumption. The effect size on attentiveness for meal composition and consuming food was medium-sized (0.43 and 0.39 respectively), the effect size for the awareness of the amounts of food selected was large (0.58) and the effect size for the decrease in alcohol consumption was small (0.19) when comparing baseline to T1. Smoking showed no effect on T1 compared to baseline.

The outcomes of participants pre COVID-19 versus during COVID-19 showed no difference on the diet related outcomes, alcohol consumption or smoking.

### Perceived fitness

The perceived fitness factors perceived health, feeling fit when waking up, feeling fit during the day and the impact of stress on daily functioning all showed an effect in the desired direction with a small effect size (between 0.05 and 0.28), except for perceived health which showed a medium effect size (0.50) at T1.

The subgroup comparison showed larger effects from baseline to T1 for participants pre COVID-19 compared to during COVID-19 on all perceived fitness factors (p < 0.02).

### Sleep and stress

The constructs sleep and stress both showed a decrease at T1 compared to baseline with a small effect (0.30 and 0.23 respectively) in the desired direction.

The outcomes of participants pre COVID-19 showed a larger reduction at T1 in stress perception compared to the outcomes of participants during COVID-19 (p < 0.01). No differences were found between both subgroups on sleep.

### Post-hoc sensitivity analysis

The post-hoc sensitivity analysis showed that on individual level, in general the trend in components related to behaviour (i.e., physical activity, diet attentiveness, sleep and stress) had a similar pattern as the trend in anthropometric outcomes except for smoking and sleep. In short, more physical active minutes, more attentiveness to diet and improved stress management are related to weight loss in CooL.

## Discussion

In this study we analysed changes in various outcomes on participants after eight months of the CooL-intervention as well as differences in outcomes between participants pre and during COVID-19. Looking at the changes in outcomes over eight months of CooL, the analyses showed positive changes compared to baseline. The largest effect sizes were found on weight, BMI, waist circumference, perceived health and diet attentiveness (i.e., attentiveness to meal composition, awareness to amounts of food and attentiveness to consuming). Changes in behaviour and perceived fitness varied between small and medium effect size, whereas changes in anthropometrics showed a medium to large effect size.

Encouraging participants to take responsibility for their personal lifestyle is an essential element of CooL. Participants prioritize their health-related behaviours and define personal actions. The consequence of this set-up is that all participants start working on a behavioural aspect of their choice, which may lead to changes that are averaged out when looking at a population level. The timeframe of this study only covers the first eight months of the intervention implying that participants might not yet have initiated changes in all health-related behavioural domains. Note that during the first eight months of the study, major changes were already found on anthropometrics and perceived health. It is plausible that these small behavioural changes together sum up to medium and large-sized changes in anthropometric outcomes and perceived health. The post-hoc sensitivity analysis gives support to the assumption that the behavioural changes correlate with changes in anthropometrics. Two exceptions are smoking and sleep: in many cases people that quit smoking, gain weight during the first few months of abstinence [37] and the relation between sleep-related behaviour and weight is likely to be more indirect (i.e., via hormonal pathways and other behaviours) [12].

The average weight loss per participant was 3.2%, with 29% of the participants losing 5% or more. This corresponds with a decrease of 1.16 points in BMI and an average decrease of 3.44 kg after the first eight months of CooL. Compared to previous research on the CooL-pilot [15], HealthyLIFE-study [16] (with respectively an average decrease in weight of 2.3 and 2.4 kg) and research on similar interventions [13, 14, 38], these results are promising. Future research on the two-year results is needed to determine the effect of the CooL-intervention on the total duration of 24 months.

The outcomes of participants pre COVID-19 versus during COVID-19 showed differences only on self-mastery (p = 0.01), sedentary time (all underlying constructs p < 0.02), perceived fitness (all underlying constructs p < 0.02) and stress (p < 0.01). The differences found are partly in line with previous research: a larger decrease of perceived stress when participating pre COVID-19, is in line with the findings of Ammar [39]. Ammar identified a negative effect on mental-wellbeing, on mood and feelings during COVID-19 [39]. Especially vulnerable populations have been found to show an increase in stress [40, 41]. For alcohol usage and smoking two opposite outcomes were seen during COVID-19: an increase due to distress or boredom and a decrease in usage linked to prevention and health withstanding the threat of COVID-19 or limited access and resources [42, 43]. On population level, increases in alcohol usage for some people even out with decreases in alcohol usage for others, leading on population level to changes in alcohol usage close to zero [42]. A similar reasoning for smoking could explain that no effect on alcohol and smoking was seen for the CooL-participants during COVID-19 [43]. However, the comparison of this intervention study in active participants with population-level observational studies should be done with great caution as participating in an intervention can trigger behaviour change on lifestyle related topics including alcohol and smoking.

There are also several findings that are not in line with previous research: firstly, research on the effect of COVID-19 on sleep in several European countries showed delayed sleep timing, more time spent in bed and impaired sleep quality [44, 45]. It also showed large individual differences in perceived sleep quality mainly depending on pre-pandemic sleep quality. In general, negative affect and feelings of worry linked to COVID-19 restrictions, were associated with changes in sleep quality [44, 45]. In contrast, the present study showed that the improvements in perceived sleep quality did not differ prior versus during COVID-19.

Secondly, other studies on the impact of COVID-19 on lifestyle-related behaviour have shown that most health behaviours were largely affected by the pandemic and its related measures. Regarding diet, Huber et al. [46] showed an increase in food consumption, especially for overweight people. Furthermore, the majority of studies have shown a decrease in physical activity and an increase in sedentary behaviour during COVID-19 lockdowns across several populations [25, 47, 48]. The CooL subgroup analysis showed no differences for both diet and physical activity between the runtime of CooL pre versus during COVID-19. The changes in sedentary time were even more desirable for participants in CooL during the pandemic. In times of a major pandemic consistency in behaviour and/or small improvements in behaviour are likely to be a huge win.

The effect of CooL on the three anthropometric outcomes was not affected by COVID-19 as the subgroup analyses showed no difference between participation in CooL pre or during COVID-19. This is a striking result given the outcomes of previous research on this topic: two studies on weight change during COVID-19 pandemic indicated an average weight gain of 1.5 to 2 kg [49, 50], whereas an online questionnaire in The Netherlands even showed an average weight gain of 5.6 kilos [51]. Overall, the results of this study indicate that the effect on the anthropometric outcomes of the CooL-participants were not affected by COVID-19. Participating in the CooL-intervention may thus have protected against relapsing to unhealthier behaviour despite a decreased sense of self-mastery and increased stress.

### Limitations and strengths

During the time of the study the questionnaire was subject to minor revisions. We intended to keep the scope of the questions and answers similar for all versions, but we cannot rule out an effect on the study outcomes. However, as with any observational study, differences in outcomes could also be due to differences in demographics, zeitgeist and the emergence of COVID-19.

The sudden emergence of COVID-19 was unforeseen and can be considered a limitation of the study as it impacted the intervention and outcomes in many ways. At the same time, it can be regarded as an opportunity to study the effects of a large-scale health promotion intervention during a pandemic.

The lack of a control group inhibits us to draw strong conclusions on the effectiveness of the intervention. Results indicate changes in outcomes over time, but inferences regarding intervention effectiveness need to be interpreted with caution.

Motivation was questioned using a scale derived from Self-Determination Theory with six questions. The exploratory factor analysis did not justify summarizing the motivational items in one construct by item score means. Consequently, we looked at these motivation items separately instead of using one summarizing construct, in line with Chemolli and Gagné [52]. However, this approach led to uninterpretable results. Anecdotal evidence collected by feedback from participants and coaches indicated that the motivational questions caused confusion and were considered difficult to interpret for participants. This led to a major revision of the measurement of this construct in a new version of the questionnaire for future data collection and research. Physical activity, diet attentiveness, smoking and alcohol use were asked in retrospective via questionnaires which entails the risk of overestimation. However, whenever possible we used multiple questions that allowed for cross-checking. In addition, we looked at the difference between T0 and T1, which probably led to an overestimation in both measurements, i.e., with less risk of overestimation in the change scores. Furthermore, we used the same measurements for these constructs in previous studies, supporting comparability.

Despite all attempts to collect additional data, we did not receive enough data to draw strong conclusions on the different ways of implementing CooL during COVID-19 (e.g., digital versus physical contact and starting in digital versus face-to-face mode) and only on whether it was implemented before or during COVID-19. In retrospect, we found that the ratio of participants who started before COVID-19 to those who started during COVID-19 was off balance, but this mainly reflects the number of participants who completed the first phase of CooL in a given period. To draw strong conclusions on the different ways of implementing CooL in digital mode, more data is needed on various implementation modes of CooL. A total of 37 participants in the overall dataset could not be assigned to the subgroups pre or during COVID-19 leading to slightly deviating average outcomes in the subsamples.

In normal conditions anthropometrics are measured by the CooL-coach in order to minimize self-report bias. As COVID-19 restrictions could have changed the measurement method, additional information was gathered from the CooL-coaches that were the main data suppliers (representing data of a quarter of the participants, n = 490). This information indicated that in general, physical measurements took place either by the coach or on a distance of 1.5 m under direct supervision of the coach.

### Future recommendations

This study provides insights on the outcomes after participating eight months in CooL and on the possible influence of COVID-19 on the outcomes, but it also provides input on recommendations for future research on CooL and adaptations to the questionnaires used for CooL:

- Validation research of the question on social support and the questions on diet attentiveness as well as the newly constructed questions on motivation, initiated by the desire to validate the measurement instruments on these constructs.
- Development of an equally effective online CooL-intervention, preserving the existing working elements and objectives of CooL as much as possible.
- Effect study of CooL after 24 months participation (including the outcomes on phase 1 and phase 2).
- Addition of the CooL questionnaire with questions on the mode of delivery of CooL (physically or digitally).

## Conclusions

After eight months of CooL, large effect sizes on changes in anthropometrics and perceived health were found, irrespective of participation during the COVID-19 pandemic. The results show that small changes in multiple behaviours go along with a large positive change in perceived health and health-related outcomes in line with the lifestyle coaching principles. Participating in the CooL-intervention may have protected against engaging in unhealthier behaviours during the pandemic, despite a decreased sense of self-mastery and increased stress.

## Data Availability

The datasets generated and/or analysed during the current study are not publicly available because the informed consent statement to using data at the individual level was limited to the authors of this article and are only available from the corresponding author on reasonable request.

## Abbreviations

BLCN: Beroepsvereniging Leefstijlcoaches Nederland
BMI: Body mass index
CBO: Centraal Begeleidingsorgaan
CLI: Combined lifestyle intervention
CooL: Coaching op Leefstijl
COVID-19: Corona virus disease 19
FHML-REC: Ethics review committee of the faculty of health, medicine and life sciences
NHG: Nederlands Huisarts Genootschap
NICE: National institute for health and care excellence
PSQI: Pittsburgh sleep quality index
RIVM: Rijksinstituut voor Volksgezondheid en Milieu
SPSS: Statistical package for the social sciences
